# A DNA aptamer efficiently inhibits the infectivity of *Bovine herpesvirus* 1 by blocking viral entry

**DOI:** 10.1038/s41598-017-10070-1

**Published:** 2017-09-18

**Authors:** Jian Xu, Xixi Zhang, Shuanghai Zhou, Junjun Shen, Dawei Yang, Jing Wu, Xiaoyang Li, Meiling Li, Xiufen Huang, Joshua E. Sealy, Munir Iqbal, Yongqing Li

**Affiliations:** 1 Institute of Animal Husbandry and Veterinary Medicine, Beijing Academy of agricultural and Forestry Sciences, Beijing, 100097 P.R. China; 2 Animal Science and Technology College, Beijing University of Agriculture, Beijing, 102206 P.R. China; 3 College of Animal Science and Technology, Jiangxi Agricultural University, Nanchang, Jiangxi 330045 P.R. China; 4 The Pirbright Institute, Ash Rd, Pirbright, Woking, GU24 0NF UK

## Abstract

Bovine herpesvirus 1 (BoHV-1) is an important pathogen of domestic and wild cattle responsible for major economic losses in dairy and beef industries throughout the world. Inhibition of viral entry plays a crucial role in the control of BoHV-1 infection and aptamers have been reported to inhibit viral replication. In this study, nine DNA aptamers that target BoHV-1 were generated using systemic evolution of ligands by exponential enrichment. Of the nine candidates, aptamer IBRV-A4 exhibited the highest affinity and specificity for BoHV-1, which bound to BoHV-1 with a Kd value of 3.519 nM and demonstrated the greatest virus binding as shown by fluorescence imaging. The neutralizing ability of aptamer IBRV-A4 was determined using neutralization assays and real time PCR in BoHV-1 infected Madin-darby bovine kidney cells. Virus titration, immunofluorescence and confocal laser scanning microscopy showed virus replication significantly decreased when aptamer IBRV-A4 was added to BoHV-1 infected MDBK cells at 0 and 0.5 hours post-infection, whereas no change was seen when IBRV-A4 was added 2 hours post-infection. This concludes that aptamer IBRV-A4 efficiently inhibits viral entry of BoHV-1 in MDBK cells and is therefore a novel tool for diagnosis and treatment of BoHV-1 infection in cattle.

## Introduction

Bovine herpesvirus 1 (BoHV-1) primarily causes infection in the upper respiratory tract (IBR) which is clinically characterized by dyspnea, hyperpyrexia, nasitis and conjunctivitis^1^. BoHV-1 can also act as a predisposing factor for vaginitis and abortions in adult cattle, and encephalitis and dystaxia in calves^2^. The world organization for animal health has placed BoHV-1 into category B for infectious diseases because of its worldwide prevalence and its significant impact on cattle industries^3^. BoHV-1 was first identified in Germany in the 19th century, before it spread from Europe to North America in the 1950s^3^. China recorded its first BoHV-1 infection in dairy cows in the 1980’s and continues to be threatened, with seropositive rates in Chinese cattle reaching 35–55% in many farms^1^. Following acute infection with BoHV-1, lifelong latency is established that generates latent carrier cattle. Reactivation of BoHV-1 in these latent carriers causes BoHV-1 to be shed and rapidly spread throughout a herd which may include infection of naïve animals^4,5^. To break this transmission cycle it is necessary to remove BoHV-1 infected latent carriers from cattle herds using diagnostic tools that offer high specificity and sensitivity. Furthermore, use of potent antiviral treatment at the acute phase of infection can potentially protect cattle from developing latent infection.

Vaccination is the most commonly used method of BoHV-1 control and is effective at reducing clinical symptoms and limiting virus shedding but does not provide complete protection against BoHV-1 transmission within cattle herds^6^. This is largely due to the virus’s ability to evade the host immune system and establish latent infection^6,7^. Antiviral drugs such as monoclonal antibodies (McAbs), interferon (IFN), co-receptor antagonists and small molecule inhibitors have also been developed that treat BoHV-1 infection by targeting pathogen structural features or host cellular receptors to block viral entry^8–10^. Many of these antiviral drugs are effective in treating virus infection^11^, however non-specificity and clinical side-effects make these options imperfect^12^. Furthermore, BoHV-1 pathogenesis and the mechanism of latency are comparable with herpes simplex sirus-1 (HSV-1) and is therefore a good model for the development of novel tools to treat and control HSV-1 infection.

Aptamers are single-stranded DNA or RNA oligonucleotides that can be selected against target ligands after multiple rounds of a selection process called systematic evolution of ligands by exponential enrichment (SELEX)^13^. Since 1990, aptamers have been selected against a wide variety of target molecules including chemical elements, biotic components, proteins and receptors^14^. Similarly to antibody-antigen affinities, aptamers can bind to target molecules based on their three-dimensional structures^15^. Aptamers have shown comparable or better affinities than their antibody counterparts and have many advantages including ease of selection, synthesis and modification, high stability and low development cost^14^. A variety of aptamers have been produced that recognize a diverse range of target molecules and pathogens including *Mycoplasma bovis*, avian influenza and herpes simplex virus^16–18^. These reports highlight aptamers as good candidates for diagnostic tools and antiviral agents^16,19^. However, there is currently no aptamer available as a therapeutic or prophylactic agent against BoHV-1.

In this study, we isolated nine DNA aptamers that target BoHV-1 from a pool of random DNA sequences using SELEX. From these nine candidate aptamers, a single aptamer henceforth referred to as IBRV-A4, was identified which binds BoHV-1 with high affinity and specificity. Neutralization activity of this aptamer was determined in BoHV-1 infected Madin-darby Bovine Kidney (MDBK) cells. Our investigation demonstrated that aptamer IBRV-A4 efficiently inhibits the infectivity of BoHV-1 by blocking viral entry.

## Results

### *In vitro* selection of ssDNA aptamers against BoHV-1

In order to maximize the enrichment of ssDNA aptamers binding to BoHV-1, BoHV-1 virus was purified through density gradient centrifugation. The ssDNA aptamers were then selected from a random DNA library by SELEX using purified BoHV-1 as an immobilized target^13^. The initial library contained approximately 10^15^ ssDNA sequences, with each sequence comprising 39–40 random nucleotides at the core region and 20–21 5′ and 3′ flanking nucleotides that made up the constant primer regions. As the cycles of selection proceeded, a decreasing concentration of target BoHV-1 was used in order to isolate a pool of aptamers with enhanced BoHV-1 specificity (Table 1). After eight rounds of SELEX, the remaining pool of aptamers were cloned and sequenced. Nine consensus sequences out of 58 clones were obtained by comparative analysis. Those with repeated frequency are listed in Table 2 and were taken forward for further characterization. These results suggested that aptamers against BoHV-1 had been well enriched and selected for, with the nine selected aptamers containing a combination of three conserved sequences: GGGTGG, GGGAGG and GGTTTG. Eight aptamers contained the sequence GGGTGG, seven aptamers contained the sequence GGGAGG and two aptamers contained the sequence GGTTTG. The latter two aptamers were IBRV-A3 and IBRV-A4 (Table 2).

**Table 1:** SELEX parameters for eight rounds of aptamer selection.

SELEX round	Incubation of BoHV-1 (μg)	Incubating time (min)	ssDNA pool (μM)
1	100	30	10
2	100	30	10
3	100	30	10
4	100	30	10
5	50	30	10
6	25	30	10
7	10	30	10
8	5	30	10

**Table 2:** Isolation and frequency of candidate aptamers after multiple rounds of SELEX.

Aptamer	Sequence of core region (5′ to 3′)^a^	No. of appearances^b^	Frequency (%)^c^	Length of variable region (nt)
IBRV-A1	GGGAGGTGGGCGGGTGTTACGTGCCACGCTTTCGTGTATG	3	5.2	40
IBRV-A2	GGGAGGTGGGTGGGCGTGCCATACGTGACGGCTACTGTGG	3	5.2	40
IBRV-A3	GTGGTCGGGGTGGGTGGTG**GGTTTG**TATTGCCTGTCGAC	3	5.2	39
IBRV-A4	GGCGGCGGGGTGGGGTGGGC**GGTTTG**ATTCCCATGGGTGC	15	25.7	40
IBRV-A5	GGGAGGCGGGTGGGGCTGCTGCACAGTGTGTTACGGTTG	3	5.2	39
IBRV-A6	GGGAGGCGGGTGGGCCATATTCGCAGATCTTTGTCTGTGC	11	19.0	40
IBRV-A7	GGGAGGCGGGTGGGTCGTCGGGGTGCGTCGTTCTGTGTGG	13	22.4	40
IBRV-A8	GGGAGGCGGGCGGGTGGCTTGCGGTCAGCGTTATGTGCGG	4	6.9	40
IBRV-A9	GGCACATTGCAGGGGAGGCGGGTGGGGATGCATCGGCCC	3	5.2	39

^**a**^Only the sequence of the central variable region is shown. The unbroken underlining and broken underlining indicate the GGGAGG and GGGTGG motifs, respectively. Boldface indicates the GGTTTG motif in IBRV-A3 and IBRV-A4. ^**b**^Frequency of each aptamer sequence among 58 total sequences. ^**c**^Number of appearances 100/58.

### Affinity and specificity of selected aptamers

**Figure 1:**
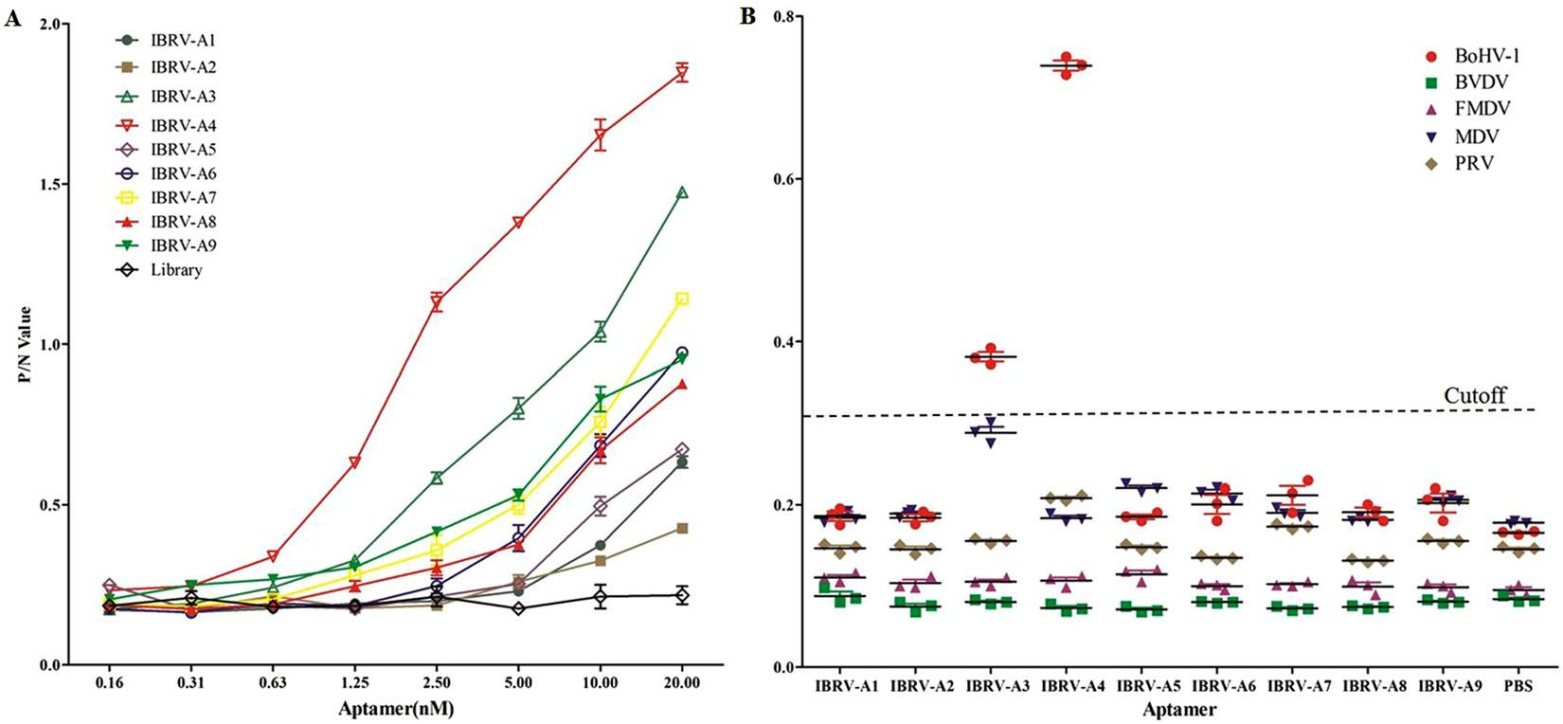
Binding affinity and specificity of selected aptamers against BoHV-1. ELONA was used to determine binding affinities (**A**) and specificities (**B**) of selected aptamers against BoHV-1. In each graph, the Y-axis represents the measurement of bound aptamer at OD_450_. Different concentrations of aptamer were used for determining binding affinity as detailed on the X-axis (**A**) and a consistent concentration of aptamer was used for determining binding specificity as detailed in the X-axis (**B**). A cutoff OD_450_ of 0.323 was determined using PBS control.

The binding affinity and specificity of selected aptamers to BoHV-1 was evaluated by enzyme-linked oligonucleotide assay (ELONA)^20^. Aptamers, designated IBRV-A1 to IBRV-A9 showed a range of affinities toward BoHV-1 and all had greater affinity compared to the Library control (Fig. 1A). We observed that the aptamer binding affinities were also dose dependent but not necessarily associated with enrichment as indicated in Fig. 1A and Table 2. ELONA showed aptamer IBRV-A4 had the greatest binding affinity out of all nine candidate aptamers. IBRV-A3 also exhibited a high level of binding affinity but had a relatively low frequency of appearance compared to the other nine aptamers. Comparative ELONA analysis showed that aptamers IBRV-A3 and IBRV-A4 had no non-specific binding to selected control viruses; control viruses used were *Alphaherpesviruses* (Marek’s disease virus and Pseudorabies virus) and two unrelated cattle viruses (Bovine viral diarrhea virus and Foot and mouth disease virus). Aptamer IBRV-A4 could bind BoHV-1 with a significantly higher OD_450_ value (Fig. 1B). These results conclude that aptamer IBRV-A4 possessed high specificity and affinity toward BOHV-1.

### Secondary structure and dissociation constant (Kd) of aptamer IBRV-A4

**Figure 2:**
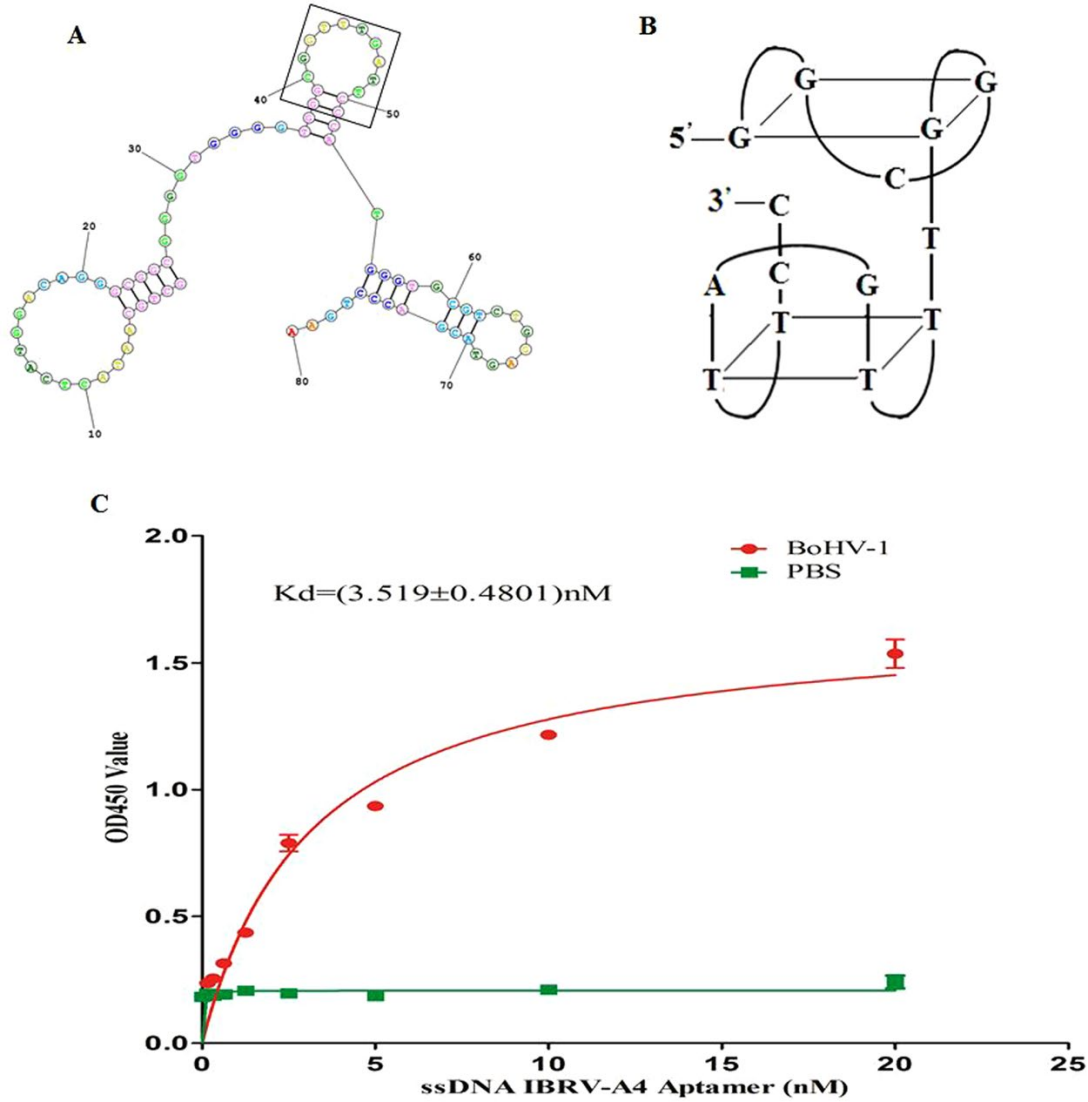
Secondary structure and dissociation constant (Kd) of aptamer IBRV-A4. The predicted secondary structure of aptamer IBRV-A4, the box highlights a loop and stem structure in the core region (**A**). One potential G-quadruple structure and one potential T-quadruple structure identified in aptamer IBRV-A4 (**B**). Dose-dependent ELONA to determine the dissociation constant (Kd) of aptamer IBRV-A4 (C).

Aptamer IBRV-A4 was shown to be the most promising candidate and therefore its secondary structure was further characterized. Two-dimensional stem-loop structures of aptamer IBRV-A4 were predicted using the tool: http://mfold.rit.albany.edu/. As shown in Table 2 and Fig. 2A, IBRV-A4 comprised an imperfect 5 base-pair stem and 16 nt loop at the 5′ end and a 7–8 base-pair stem and 7 nt loop at the 3′ end. These were linked by a 9 nt motif: GGGGTGGGG and a 4 base-pair stem and 10 nt loop: CGGTTTGATT. In addition, there was a unique single loop in the core region between nucleotides 38 and 51 (box region), suggesting that there was one potential G-quadruplex structure and one potential T-quadruplex structure (Fig. 2B).

To confirm binding affinity between aptamer IBRV-A4 and BoHV-1, the dissociation constant (Kd) was measured by ELONA. The resulting Kd values showed aptamer IBRV-A4 bound BoHV-1 at 3.519 ± 0.481 nM, suggesting that aptamer IBRV-A4 had stronger binding affinity for BoHV-1 than any other aptamer reported elsewhere (Fig. 2C)^17^.

### Aptamer IBRV-A4 bound to BoHV-1 infected MDBK cells

**Figure 3:**
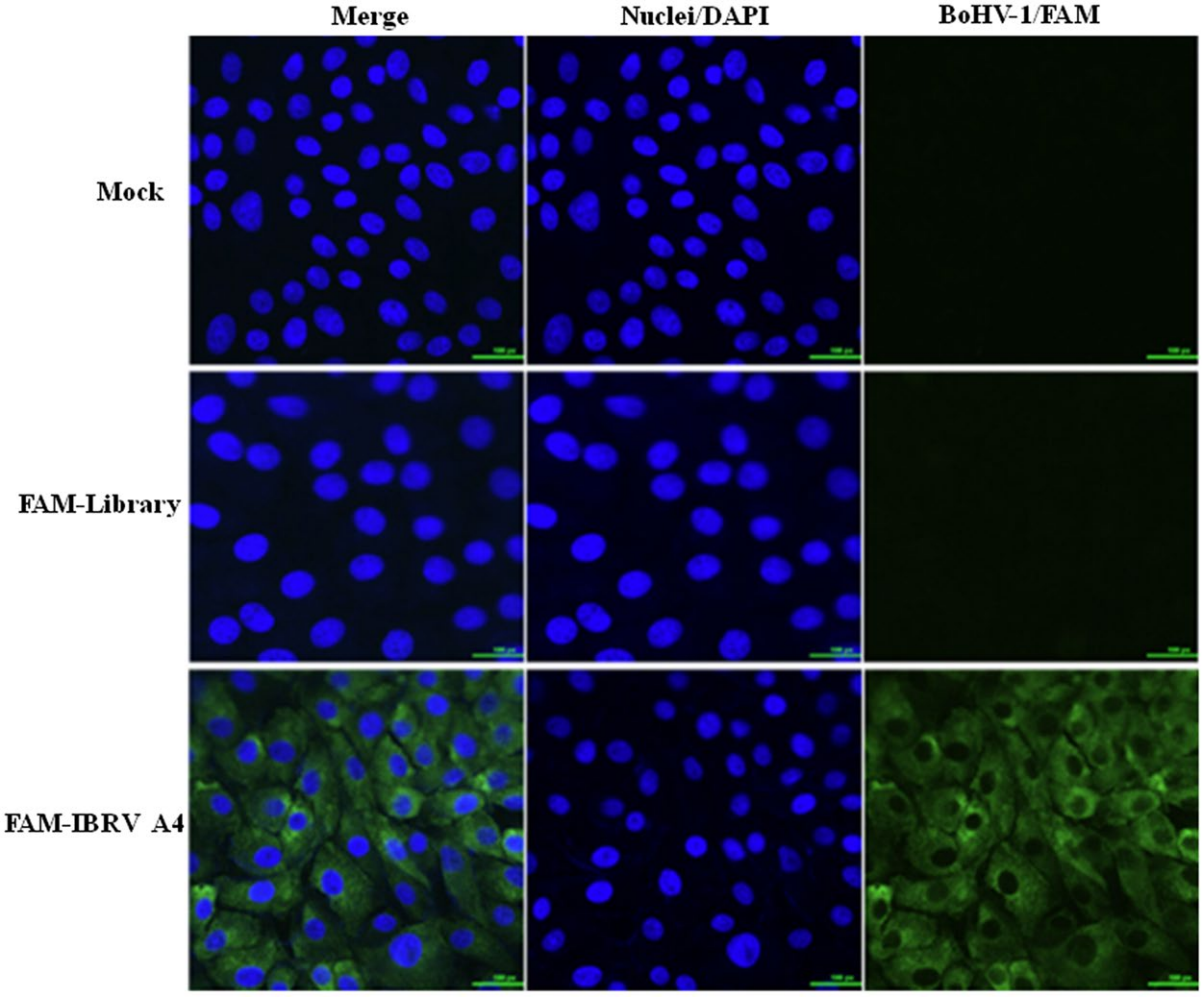
Detection of BoHV-1 in infected cells using aptamer IBRV-A4. FAM-labeled aptamer IBRV-A4 was used in fluorescence assays to determine aptamer localization in infected cells. FAM is shown in green and cellular nuclei were counterstained with DAPI shown in blue. The mock was uninfected MDBK cells treated with FAM-labeled IBRV-A4 and the FAM-Library refers to the FAM-labeled library control used to show non-specific binding by aptamers. Magnification = 200x.

Diagnostic tests could benefit from aptamers that bind specifically to virus infected cells. To explore the practical use of aptamer IBRV-A4 in the detection of BoHV-1 infected cells, we labeled aptamer IBRV-A4 and the original DNA library with fluorescein amidite (FAM) at the 5′ terminus and established a detection method similar to an immunofluorescence assay (IFA). In this assay, FAM-labeled IBRV-A4 was shown to bind BoHV-1 infected MDBK cells by presenting green fluorescence on infected MDBK cells. To exclude non-specific binding, FAM-labeled IBRV-A4 was incubated with uninfected MDBK cells and the FAM-labeled DNA library was incubated with BoHV-1 infected MDBK cells. No green fluorescence was detected in uninfected MDBK cells and only background fluorescence was observed in BoHV-1 infected MDBK cells incubated with the FAM-labeled DNA library. These results suggest that aptamer IBRV-A4 specifically bound to BoHV-1 infected cells (Fig. 3).

### Aptamer IBRV-A4 inhibits infectivity of BoHV-1

We further analyzed aptamer IBRV-A4’s ability to block BoHV-1 infectivity in MDBK cells using micro neutralization test. Serial dilutions of aptamer IBRV-A4 were mixed with BoHV-1 at 100 TCID_50_ and incubated at 37 °C for 1 hour. Aptamer-virus mixtures were inoculated onto MDBK cells in 96-well format and cytopathic effect (CPE) recorded at 72 hours post-infection. The lowest concentration of aptamer IBRV-A4 that protected MDBK cells from BoHV-1 induced CPE was 0.625 nM. The neutralization titer was calculated using the Karber method and shown to be 1:55 (Table S1)^21^.

**Figure 4:**
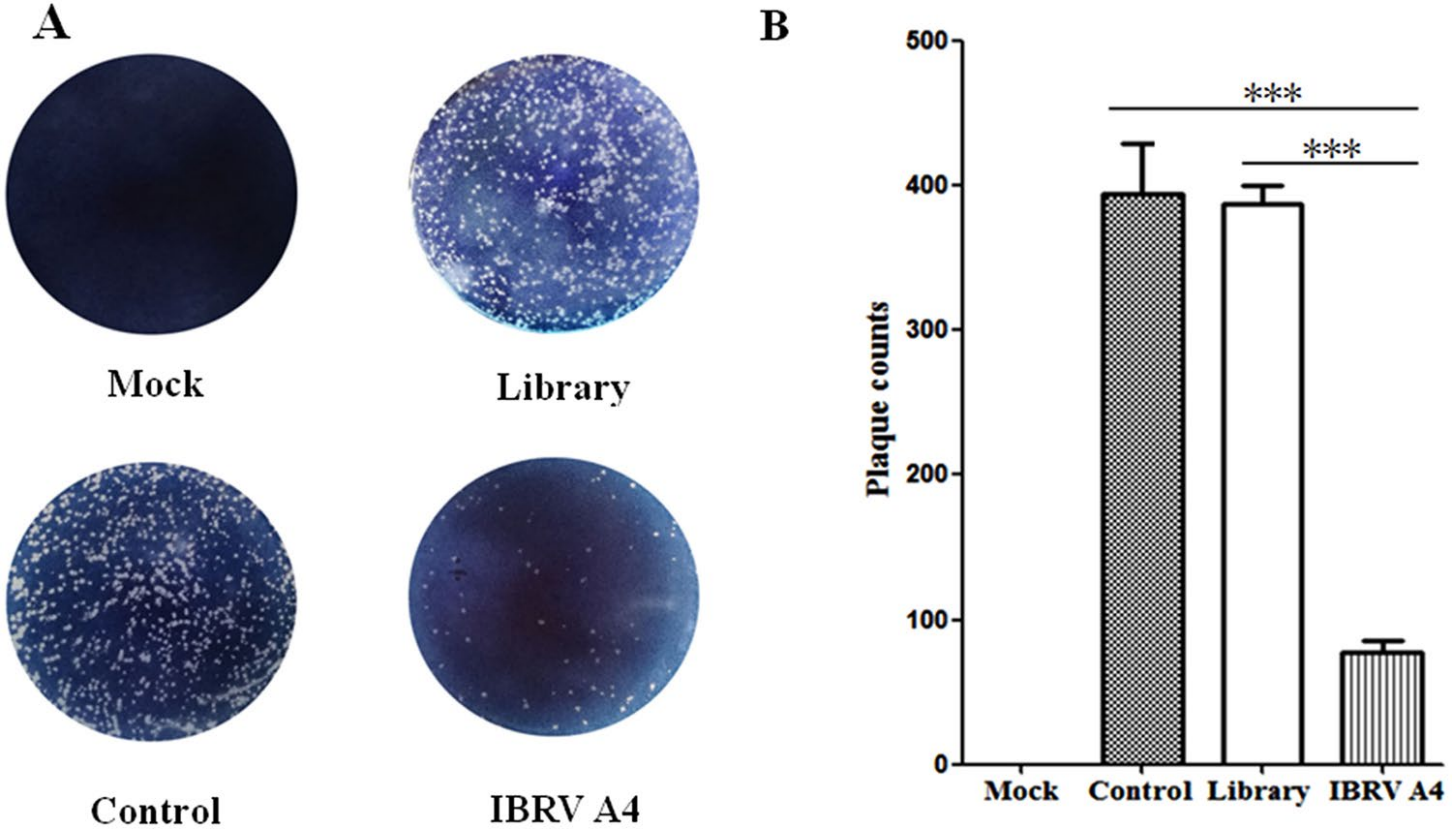
Aptamer IBRV-A4 inhibits plaque formation in MDBK cells. Plaques developed in MDBK cells after the addition of different treatments (**A**). MDBK cells were treated with either PBS (Mock), BoHV-1 only (Control), DNA library (Library) or aptamer IBRV-A4 (IBRV-A4). Plaques were stained using 0.1% toluidine blue in saline. The data was analyzed using SPSS software and represented in (**B**). “***”indicated statistically significant difference (p < 0. 001).

The antiviral activity of aptamer IBRV-A4 against BoHV-1 was also analyzed by plaque reduction neutralization test (PRNT)^22^. The plaque titer of BoHV-1 was significantly reduced (P < 0.001) in IBRV-A4 treated MDBK cells compared to DNA library treated MDBK cells (Fig. 4). These results conclude that aptamer IBRV-A4 could act as an antiviral agent by reducing BoHV-1 infectivity of MDBK cells.

### Aptamer IBRV-A4 blocks viral entry of BoHV-1 in MDBK cells

**Figure 5:**
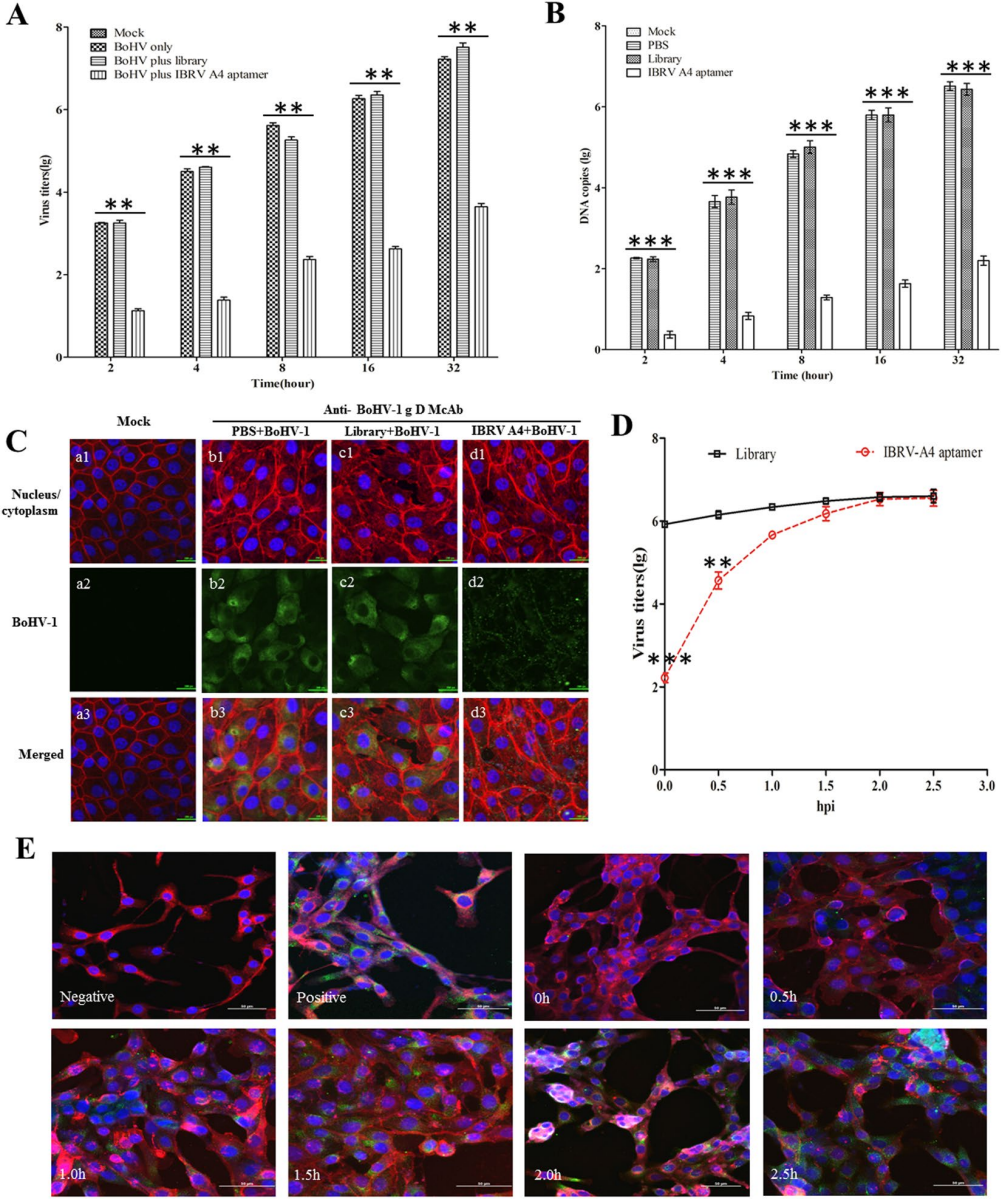
Aptamer IBRV-A4 blocks entry of BOHV-1 into MDBK cells. (**A**) Virus titers of BoHV-1 were determined after treatment with DNA library or aptamer IBRV-A4 at 2, 4, 8, 16 and 32 hours post-infection. Controls were BoHV-1 only or mock. The statistical differences between treated and control groups were determined and analyzed using SPSS software. “**” indicated statistically significant difference (p < 0. 01). (**B**) Virus replication of BoHV-1 in MDBK cells was determined by real-time PCR after treatment with DNA library or aptamer IBRV-A4 at 2, 4, 8, 16 and 32 hours post-infection. Controls were either untreated MDBK cells (negative) or MDBK cells treated with a mixture of DNA library (or PBS) and BoHV-1 (positive). “***” indicated statistically significant difference (P < 0. 001). (**C**) Laser confocal microscopy showed that aptamer IBRV-A4 inhibited BOHV-1 infection by perturbing viral entry into MDBK cells. The Mock represented uninfected cells (a1-a3), MDBK cells infected with BOHV-1 and pre-treated with PBS (b1-b3), DNA library treated cells (c1-c3) and aptamer IBRV-A4 treated cells (d1-d3). Magnification = 200x. (**D**) MDBK cells infected with 100 TCID50 of BoHV-1 and treated with 0.625 nM of DNA library or 0.625 nM of aptamer IBRV-A4 at 0, 0.5, 1, 1.5, 2 and 2.5 hours post-infection. BoHV-1 titers from each time point were determined. The black line represents BoHV-1 infected MDBK cells treated with DNA library. The red dotted line represents BoHV-1 infected MDBK cells treated with aptamer IBRV-A4. “***” indicated statistically significant difference (p < 0. 001), “**” indicated statistically significant difference (p < 0. 01). (**E**) MDBK cells grown on coverslips and infected with 100 TCID50 of BoHV-1, then treated with 0.625 nM of DNA library or 0.625 nM of aptamer IBRV-A4 at 0, 0.5, 1, 1.5, 2 and 2.5 hours post-infection. BoHV-1 in MDBK cells was detected by IFA based on anti-BOHV-1 gD Monoclonal antibody. MDBK cell membrane was stained with DiI (1,1′-Dioctadecyl -3,3,3′,3′-tetramet hylindocarbocyanine perchlorate). Cellular nucleus was stained with DAPI. Cells were observed under laser confocal microscopy. BoHV-1 in MDBK cells is shown in green, cell membrane is shown in red and nuclei are shown in purple. Magnification = 200x.

To understand the mechanism by which IBRV-A4 protects MDBK cells from BoHV-1 infection, BoHV-1 from infected MDBK cells was titrated by TCID_50_ at 2, 4, 8, 16 and 32 hours post-infection. BoHV-1 infected cells that were treated with either the DNA library control or PBS mock control showed increasing virus titers as infection proceeds. Virus titers from IBRV-A4 treated cells showed ~100 fold less virus compared to controls from 2 hours post-infection and was sustained for the duration of the experiment (P <0.01). Notably, no statistically significant increase in virus titer was seen even at 32 hours post-infection in cells treated with IBRV-A4 prior to BoHV-1 infection (Fig. 5A). This was also in agreement with the real time PCR data showing replication of BoHV-1 in MDBK cells (Fig. 5B), which implied that aptamer IBRV-A4 played a role in the early phase of BoHV-1 infection. In order to show IBRV-A4 protected MDBK cells by blocking viral entry, an immunofluorescence assay involving a McAb that targets the gD protein of BoHV-1 was employed. Confocal microscopy observations within the first 2 hours of infection showed viral gD-specific fluorescence localized to the membrane of DNA library treated and PBS mock treated MDBK cells. Very weak fluorescence was shown on cells treated with aptamer IBRV-A4 (Fig. 5C). Virus titers significantly decreased (p < 0.01) when aptamer IBRV-A4 was added to infected MDBK cells at 0 and 0.5 hours post-infection. However, there was no significant difference (p > 0.05) in replication of BoHV-1 in MDBK cells when IBRV-A4 was added after 2 hours post-infection, compared with the DNA library control (Fig. 5D). These findings were consistent with the confocal microscopy observations that there was no BoHV-1 specific green fluorescence in the BoHV-1 infected MDBK cells treated with 0.625 nM of IBRV-A4 post-infection at 0 and 0.5 hours (Fig. 5E). However, BoHV-1 specific fluorescence was distributed increasingly inside the cells treated with aptamer IBRV-A4 after 2 hours post-infection. Taken together, these results conclude that aptamer IBRV-A4 disrupted viral entry into MDBK cells thus protecting them from BoHV-1 induced CPE.

## Discussion

As one of the most common pathogens in cattle, BoHV-1 infection has attracted more and more attention from scientists and health officials around the world, and this includes China. Various diagnostics and vaccines are available for the control of BoHV-1^23,24^ However, no strategy is able to completely eradicate and control BoHV-1 infection in cattle due to limitations in antivirals and the mechanism of viral latency in infected animals^25,26^. Therefore, it is desirable to seek novel diagnostic tools and antiviral agents that treat and control BoHV-1 infection in latent carrier cattle. Aptamers have been reported to specifically bind and inhibit target viruses and have the potential to become versatile tools in the face of viral infection^27–29^. In this study, nine candidate ssDNA aptamers targeting BoHV-1 were identified from a library of random oligonucleotides using 8 rounds of SELEX against purified immobilized BoHV-1. Of the nine aptamers, IBRV-A4 exhibited the greatest binding affinity and specificity for BoHV-1 (Fig. 1). This aptamer was taken forward for further characterization.

Aptamers are able to form three-dimensional structures and their secondary structures influence their biological properties^30^. Stem-loop patterns in aptamers significantly influence binding affinities^31^. In this study, we identified conserved regions among candidate aptamers which are not typically seen. Eight of these aptamers contained GGGTGG, seven aptamers contained GGGAGG and aptamers IBRV-A3 and IBRV-A4 contained GGTTTG which indicated that that a range of aptamers targeting the same pathogen are able to exhibit different structures and characteristics, as seen previously^14,15^. In our study, aptamer IBRV-A4 had a unique single loop structure in the core region between nucleotides 38 and 51 which contained one potential G-quadruplex structure and one potential T-quadruplex structure (Fig. 2 A,B). These structures have been shown to play an important role in the biological function of aptamers but their mechanism of action is not yet fully understood^32^. The dissociation constant (Kd) between BOHV-1 and candidate aptamers was used to measure binding affinities, which has been used previously^29^. In this study, IBRV-A4 bound to BoHV-1 with a Kd value of 3.519 ± 0.4801 nM (Fig. 2C) which indicated that aptamer IBRV-A4 had a strong binding affinity for BoHV-1. In order assess the potential of this aptamer as a tool for BoHV-1 detection, aptamer IBRV-A4 was labeled with FAM at the 5’ terminus and applied to show that FAM-labeled aptamer IBRV-A4 could detect BoHV-1 infected MDBK cells through localization of fluorescence on the membrane of infected cells. In contrast, there was no fluorescence in uninfected MDBK cells and very weak fluorescence in infected MDBK treated with FAM-labeled DNA library control, suggesting aptamer IBRV-A4 specifically recognizes BoHV-1 in MDBK cells. Therefore, it was predicted that aptamer IBRV-A4 had neutralization activity similar to antibodies against BoHV-1 and thus might act as a novel reagent to detect BoHV-1 infection.

In many cases, aptamers possessing neutralizing activity have been regarded as important components of antiviral strategies^27,29,32^ and have been clinically used to treat viral infection^14,33^. In this study aptamer IBRV-A4 had a neutralization titre of 1:55 and was shown to efficiently inhibit multiplication of BoHV-1 in MDBK cells as shown by plaque reduction assay (Fig. 4). Furthermore, we found that BoHV-1 treated with aptamer IBRV-A4 prior to inoculation of MDBK cells reduced the viral titre 100-fold compared to virus alone (Fig. 5A and B) which implied that aptamer IBRV-A4 played an inhibitory role in the initial phase of BoHV-1 infection. Immunofluorescence assays showed BoHV-1 specific fluorescence localized to the membrane of infected MDBK cells in the first 2 hours of infection and very weak BoHV-1 specific fluorescence was seen on MDBK cells treated with aptamer IBRV-A4 prior to virus inoculation (Fig. 5C). This strongly suggests that aptamer IBRV-A4 binds to BoHV-1 to neutralize virus and prevent BoHV-1 from establishing infection in MDBK cells. In previous reports, aptamers against influenza virus suppressed virus proliferation by preventing viral attachment to host cells at the initial stage of viral infection^28,34^. To verify that our aptamer IBRV-A4 had a similar ability, we titrated virus from infected cells from different time points post-infection and treated with aptamer IBRV-A4. We showed that aptamer IBRV-A4 had a significant effect on replication of BoHV-1 in MDBK cells when added within the first 1.5 hours after infection (Fig. 5D). These findings were consistent with IFA and confocal microscopy observations (Fig. 5E). The above results showed that aptamer IBRV-A4 blocked viral infection of BoHV-1 in MDBK cells during the first 2 hours of infection by blocking virus entry in MDBK cells.

In summary, our study initially isolated a set of candidate aptamers against BoHV-1 which were taken forward for further characterization. Aptamer IBRV-A4 was shown to bind BoHV-1 with the highest affinity and specificity as indicated by a Kd of 3.519 nM and by fluorescence imaging. The micro neutralization assays and plaque reduction assays as well as real-time PCR analysis demonstrated that aptamer IBRV-A4 could inhibit replication of BoHV-1 in MDBK cells. We confirmed that aptamer IBRV-A4 blocked adsorption of BoHV-1 to MDBK cells at the initial phase of infection thus neutralizing BoHV-1 infectivity. To conclude, aptamer IBRV-A4 is a promising candidate that could be developed as a tool in diagnosis and treatment of BoHV-1 in cattle.

## Material and Methods

### ssDNA library and viruses

The ssDNA library and PCR amplification primers were synthesized by Shanghai Sangon Biotech Co. Ltd. (Shanghai, China). The ssDNA library sequences contained a random 40 nt central domain and flanking primer binding regions of 20 nt. The sequence layout for the ssDNA oligonucleotides was as follows: 5′-GCTGCAATACTCATGGACAG-(central domain of 40 random nts)-GTCTGGAGTACGACCCTGAA-3′. Primer FP: 5′-GCTGCAATACTCATGGACAG-3′, primer BP: 5′-TTCAGGGTCGTACTCCAGAC-3′. Viruses used in this study are listed in Table 3 and were produced as follows: BoHV-1 and BVDV were grown in MDBK cells, MDV was grown in chick embryo fibroblasts, PRV was grown in BHK21 cells, and inactivated FMDV antigen was acquired from Lanzhou veterinary research institute, Chinese academy of Agricultural sciences.

**Table 3:** Viruses used in this study.

Stain	Source
Bovine herpesvirus 1 (BoHV-1)	BK1952, from China veterinary culture collection center (CVCC), Beijing, China.
Marek’s disease virus (MDV)	RB1B, preserved in Institute of Animal Husbandry and Veterinary Medicine, Beijing Academy of agricultural and Forestry Sciences, Beijing, China.
Pseudorabies virus (PRV)	Field isolate from aborted fetuses in swine in China.
Bovine viral diarrhea virus (BVDV)	From China veterinary culture collection center (CVCC), Beijing, China.
Foot and mouth disease virus (FMDV)	Inactivated, from Lanzhou veterinary research institute, Chinese academy of Agricultural sciences, Lanzhou, China.

### Selection of candidate ssDNA aptamers

A BoHV-1 field isolate from China (Table 3) was cultured in MDBK cells in our laboratory and employed as ‘Bait’ in the SELEX assay which was adapted from Ping *et al*.^17^. Briefly, BoHV-1 was purified by density gradient centrifugation and dissolved in 100 μl of 0.05 M carbonate-bicarbonate buffer (pH 9.6) then immobilized at a concentration of 100 μg in a 96-well microplate at 4 °C overnight. Plates were then washed three times in PBST (Phosphate-buffered saline, 0.05% v/v Tween 20) and incubated in 5% bovine serum albumin (BSA)/PBST for 2 hours at 37 °C. Plates were again washed three times in PBST and then 150 μl of 10 μM ssDNA pool added to wells containing immobilized BoHV-1. This was incubated at 37 °C for 1 hour. Unbound ssDNA was washed away with PBST and ssDNA that had bound to BoHV-1 was eluted using elution buffer at 80 °C for 10 minutes (20 Mm Tris-HCl, 4 M guanidinium isothiocyanate, 1 mM dithiothreitol, pH 8.3). Bound ssDNA was isolated from the eluted solution using the DNA gel extraction kit (BioTeke Technologies Inc. China) according to the manufacturer’s instructions. Bound ssDNA was amplified by symmetry PCR (5 minutes at 94 °C, followed by 20 cycles of 20 seconds at 94 °C, 20 seconds at 60 °C, 30 seconds at 72 °C, 5 minutes at 72 °C and 5 minutes at 95 °C). PCR products were purified using the DNA gel extraction kit. The purified PCR products were used in an additional PCR to generate an enriched ssDNA library (Primers FP17 and BP17 at a ratio of 1:10; 5 minutes at 95 °C, followed by 30 cycles of 20 seconds at 95 °C, 20 seconds at 57 °C, 30 seconds at 72 °C, 5 minutes at 72 °C and 5 minutes at 95 °C). This enriched library was used in the next round of SELEX, selection parameters for each round are shown in Table 1. After eight rounds of SELEX, the remaining ssDNA fragments were cloned and sequenced. By comparative analysis of sequences, the aptamers showing the greatest frequency were selected from bacterial colonies and synthesized by Sangon Biological Engineering Technology and Services.

### Enzyme-linked oligonucleotide assay to measure binding affinity and specificity of selected aptamers

The binding affinities between aptamers and BoHV-1 were determined by ELONA^20^. Purified BoHV-1 was diluted to 1 µg/ml in selection buffer (0.05 M Na_2_CO_3_, 0.05 M NaHCO_3_, pH 9.6) and 100 μl of this was incubated in a 96-well microtiter plate at 4 °C overnight. The next day, plates were washed three times in selection buffer and blocked with 3% BSA. 100 ul of the nine candidate aptamers were denatured, biotinylated (20 nM) (Table 2) and diluted in selection buffer and added to each well at 37oC for 1 hour. Next, unbound ssDNA was washed away with three washes of selection buffer. Horseradish peroxidase labelled streptavidin (HRP-SA) (GE Healthcare) was added to each well and incubated at 37 °C for 30 minutes. Plates were washed four times and developed using substrate TMB solution (Sigma) at 37 °C for 30 minutes and then the reaction was stopped with 2 M sulfuric acid. The optical densities at 450 nm were read using a microplate reader (Biorad).

The binding specificity of aptamers was also confirmed by ELONA with some alterations to the protocol. Briefly, purified virus (Table 3) was diluted to 1 µg/ml in selection buffer and 100 μl of each virus used to coat 96-well microplates at 4 °C overnight. Plates were blocked with 3% BSA then 100 μl of 0.625 nM biotin-aptamers (Table 2) were added into each well and incubated at 37 °C for 1 hour. Plates were developed using substrate TMB solution and optical densities at 450 nm were read using a microplate reader (Biorad). Statistical analysis (ANOVA) was carried out using SPSS software version 18.0 and best fit curves at various concentrations of aptamer were modelled using GraphPad Prism 5.0. The cut off value was calculated using negative controls (PBS) using the formula: mean of OD_450_ value plus 5 times the standard deviation (5 SD).

### Fluorescence assay

MDBK cells were seeded on cover slips in 6 well plates and cultured until 80% confluent at 37 °C. Purified BoHV-1 was added to MDBK cells and incubated for a further 18–24 hours. Infected cells were fixed with 4% paraformaldehyde, permeabilized with 0.1% Triton X-100 for 1 hour, blocked with 3% BSA and incubated with FAM labeled aptamer or FAM labeled DNA library at 37 °C for 1 hour. MDBK cell nuclei were counterstained with DAPI (blue) and cells examined using a fluorescence microscope (Leica EL 6000).

### Structure prediction and measurement of dissociation constants of selected aptamers

The secondary structure of selected aptamer IBRV-A4 was predicted by Mathews Lab Web Servers^32^. The dissociation constant (Kd) of aptamer IBRV-A4 was measured by ELONA as previously described with some modifications^20^. The 96-well microplates coated with BOHV-1 were blocked with 3% BSA, serial dilutions of bio-aptamer (100 nM, 50 nM, 25 nM, 10 nM, 1 nM, 5 nM, 0 nM) were added into the wells and incubated at 37 °C for 1 hour. HRP-SA was added to each well and plates were incubated at 37 °C for 30 minutes. As with the ELONA assay described above, the optical densities at 450 nm were determined and the equation Y = BmaxX/(Kd + X) used to obtain the saturation curve and Kd between IBRV-A4 and BoHV-1 using GraphPad Prism 5.0. The Y value represented the mean value of OD_450_, Bmax is the maximal value of OD_450_ and X is the concentration of the biotinylated aptamer (bio-aptamer).

### Microtitre neutralization test

The neutralization titer of selected aptamers was measured by Microtitre neutralization test (MNT). The MNT was conducted according to a previously described protocol^35^. Briefly, MDBK cells were seeded in a 96-well plate one day prior to infection. Serial diluted aptamer IBRV-A4 (Table S1) was incubated with BoHV-1 (100 TCID50) at 37 °C for 1 hour. Virus-aptamer mixtures were inoculated onto MDBK cell monolayers and CPE was recorded after 72 hours. Survival of BoHV-1 infected cells was calculated using the dilution at which CPE was not observed. The neutralization titers were calculated using the Karber method and expressed as TCID_50_.

### Plaque reduction test

The antiviral activity of aptamer IBRV-A4 was further evaluated by plaque reduction test (PRT) according to a previously described protocol^36^. 0.625 nM of aptamer IBRV-A4 or DNA library control were mixed with an equal volume of BoHV-1 (100 TCID50) and incubated at 37 °C, 5% CO_2_ for 1 hour. 1 ml of virus-aptamer/control mixture was inoculated onto a confluent monolayer of MDBK cells in a six-well plate in triplicate. Plates were incubated at 37 °C, 5% CO_2_ for 1.5 hour with intermittent rocking. An agarose overlay was added to the infected cell monolayer and allowed to solidify at room temperature then inverted upside down and incubated for 48 hours. When viral plaques became visible, cells were fixed with 4% formaldehyde, stained with 0.1% toluidine blue in saline solution and plaques counted visually. The data was analyzed using SPSS software. P values < 0.01 were considered statistically significant.

### Assay for the inhibition of viral entry by aptamers

BoHV-1 at 100 TCID50 was incubated with 0.625 nM of aptamer IBRV-A4 or 0.625 nM of DNA library control at 37 °C for 1 hour. These mixtures were individually inoculated onto a monolayer of MDBK cells in six-well plates. The treated MDBK cells were cultured for 2, 4, 8, 16 and 32 hours, untreated MDBK cells were used as a negative control and MDBK cells treated with BoHV-1 only were used as a positive control. Supernatants from each well were harvested and used to measure the TCID_50_ at each time point. The above experiments were carried out in triplicate and the data was analyzed using SPSS software. P values < 0.01 were considered statistically significant.

To determine whether aptamer IBRV-A4 blocked viral entry into cells at an early stage, a similar experimental set up detailed above was used. BoHV-1 infected cells were treated with 0.625 nM of aptamer IBRV-A4 or 0.625 nM of the DNA library control at 0, 0.5, 1, 1.5, 2 and 2.5 hours post-infection. Supernatants were harvested and used to measure TCID_50_. Experiments were carried out in triplicate and best fit curves at various concentrations of aptamer were modelled using GraphPad Prism 5.0.

### Immunofluorescence assay

MDBK cells were seeded onto coverslips in six-well plates and cultured for 18–24 hours then treated with either aptamer IBRV-A4, the DNA library control or PBS mock control and infected with BoHV-1 for 1–2 hours. Cells were fixed with 1% paraformaldehyde and permeabilized with 0.1% TritonX-100 for 1 hour and blocked with 3% BSA. Monoclonal antibody against the gD protein of BOHV-1 and FITC-conjugated goat anti-mouse IgG (green) were added to cells. Cellular F-actin was stained with Alexa Fluor 555-conjugated phalloidin (red). Nuclei were counterstained with DAPI (blue). The cells were then observed using a confocal laser scanning microscope (Leica).

To further observe how aptamer IBRV-A4 could inhibit the infectivity of BOHV-1 at the initial phase of infection, MDBK cells were seeded on coverslips in six-well plates and inoculated with 100 TCID_50_ of BOHV-1. 0.625 nM of aptamer IBRV-A4 was added at 0, 0.5, 1, 1.5, 2 and 2.5 hours post-infection. Virus was permitted to grow for 18 hours at 37 °C, 5% CO_2_. IFA utilizing a mAb against the gD protein of BoHV-1 was used as above. MDBK cell membranes were stained with DiI (1,1′-Dioctadecyl -3,3,3′,3′-tetramethylindocarbocyanine perchlorate) and nuclei were counterstained with DAPI. The cells were observed under confocal laser scanning microscopy (Leica).

### Real-time PCR assays

To analyze the replication of BoHV-1 in MDBK cells, 100 TCID50 of BoHV-1 was incubated with 0.625 nM of aptamer IBRV-A4 or 0.625 nM of DNA library control at 37 °C for 1 hour. These mixtures were inoculated onto MDBK cells and supernatants harvested at 2, 4, 8, 16 and 32 hours post-infection. Untreated cells and PBS mock treated infected cells were used as negative and positive controls, respectively. DNA was extracted from harvested supernatant using the DNeasy Blood and Tissue Kit (Qiagen Inc.,Valencia, CA, USA). The amount of viral DNA was determined by real-time PCR by amplifying a 200 bp fragment of viral gB gene with the forward primer (5′-TCAAGTATATGTCGCTCGT GT-3′) and reverse primer (5′-TCAGCCGGGTCGCCAGCA-3′) and a Taqman probe (FAM5′-TCGCC TTGTGCTCTTGCCGCT-3′ BHQ1). Cycling conditions were 95 °C for 10 minutes; 95 °C for 30 seconds, 56 °C for 30 seconds, 40 cycles used). Ct values greater than 35 were chosen as a cut-off and samples showing higher Ct values were considered negative. Each sample was analyzed in triplicate and the data was analyzed using SPSS software. P values < 0.001 were considered statistically significant.

## Electronic supplementary material

Table S1.
