# Supplementary material for: A DNA aptamer efficiently inhibits the infectivity of Bovine herpesvirus 1 by blocking viral entry
Source: Sci Rep. 2017 Sep 18;7:11796. doi: 10.1038/s41598-017-10070-1 (PMC5603541; doi:10.1038/s41598-017-10070-1)
Supplement: Supplementary file 1 — Table S1. [file 41598_2017_10070_MOESM1_ESM.pdf]

A DNA aptamer efficiently inhibits the infectivity of *Bovine herpesvirus* 1 by blocking viral entry

Jian Xu[a,#], Xixi Zhang[a,b,#], Shuanghai Zhou[b], Junjun Shen[a],Dawei Yang[a], Jing Wu[a,c], Xiaoyang Li[a,c], Meiling Li[a], Xiufen Huang[a], Joshua E. Sealy[d], Munir Iqbal[d]＆Yong qing Li[a,*]

a. Institute of Animal Husbandry and Veterinary Medicine, Beijing Academy of agricultural and Forestry Sciences, Beijing, P. R. China, 100097

b.Animal Science and Technology College, Beijing University of Agriculture, Beijing, P. R.China, 102206

c.College of Animal Science and Technology, Jiangxi Agricultural University, Nanchang, Jiangxi, P. R. China, 330045.

d. The Pirbright Institute,Ash Rd, Pirbright, Woking, GU24 0NF, Unite kingdom

*Corresponding author: chunyudady@sina.com

#these authors contributed equally to this work.

Tel:+86-10-51503195 Fax: +86-10-51503195

Table S1. Microtitre neutralization test of aptamer IBRV-A4.

Dilution of IBRV-A4	Rate of CPE	Number of CPE	Number of survival	Total			
				Number of CPE	Number of survival	Rate of CPE	Rate of Survival (%)
2^{-1}	0/8	0	8	0	42	0/42	100
2^{-2}	0/8	0	8	0	34	0/34	100
2^{-3}	0/8	0	8	0	26	0/26	100
2^{-4}	0/8	0	8	0	18	0/18	100
2^{-5}	2/8	2	6	2	10	2/12	83
2^{-6}	4/8	4	4	6	4	6/10	40
2^{-7}	8/8	8	0	14	0	14/14	0
2^{-8}	8/8	8	0	22	0	22/22	0
